# An Observational Study on Arrhythmia During Cesarean Section Under Spinal Anesthesia: Incidence, Risk Factors, and Effects on Immediate Post-delivery Neonatal Outcome

**DOI:** 10.7759/cureus.16898

**Published:** 2021-08-05

**Authors:** Priyanka Dev, Prakash Deb, Rituparna Das, Prithwis Bhattacharyya, Nalini Sharma, Tushar Majumdar

**Affiliations:** 1 Anesthesiology and Critical Care, North Eastern Indira Gandhi Regional Institute of Health & Medical Sciences, Shillong, IND; 2 Obstetrics and Gynaecology, North Eastern Indira Gandhi Regional Institute of Health & Medical Sciences, Shillong, IND

**Keywords:** arrhythmia, cesarean section, spinal anesthesia, incidence, risk factors, uterine manipulation, neonatal outcome

## Abstract

Introduction: Various types of arrhythmia have been reported during cesarean section under spinal anesthesia. But the possible causative factors and the effects of arrhythmia on immediate post-delivery neonatal outcome are not well established.

Methods: This prospective observational study was conducted over a period of one year in a tertiary care hospital on women undergoing cesarean section under spinal anesthesia. The objectives of the study were to determine the incidence of arrhythmia, its types, the possible factors influencing arrhythmia, and the immediate post-delivery neonatal outcome. Data collected were analyzed using Statistical Package for the Social Sciences (SPSS) software version 21 (IBM Corp. Armonk, NY).

Results: In our study, the incidence of arrhythmia was 31.9% during cesarean section under spinal anesthesia; and sinus bradycardia was the most common type. Arrhythmia occurred more in women with hypotension, when maximum block height was above T_4 _level and dose of intrathecal hyperbaric bupivacaine was more than 2.2 mL (P value <0.05). Also, uterine manipulation led to sudden bradycardia and transient cardiac asystole in two patients which was preceded by subjective symptoms of pain and discomfort. None of the neonates required cardiopulmonary resuscitation or neonatal intensive care unit admission within an hour of birth. APGAR (Appearance (skin color), Pulse (heart rate), Grimace (reflex irritability), Activity (muscle tone), and Respiration) scores at 1 and 5 minutes were similar in all the newborns born to mothers with or without arrhythmia.

Conclusion: The occurrence of arrhythmia during cesarean section under spinal anesthesia, though very common, is rarely life-threatening. Keeping maximum level of block height between T_4 _and T_6, _using lower possible drug dose to provide adequate level of sensory block, prompt management of hypotension, and strict monitoring during uterine manipulation may reduce the overall incidence of arrhythmia. Intraoperative arrhythmia, however, does not adversely affect the immediate post-delivery neonatal outcome.

## Introduction

Obstetric anesthesia is one of the areas of anesthetic practice where an anesthesiologist faces many challenges owing to the care of both the mother and fetus simultaneously.

Cesarean section can be performed under general anesthesia, epidural anesthesia, or spinal anesthesia [1]. However, general anesthesia for cesarean section is rarely performed due to the increased incidence of maternal mortality [2]. Spinal anesthesia is now considered as the technique of choice. But there are observations of arrhythmia and even unexpected cardiac arrests under spinal anesthesia [3-5]. Different types of arrhythmias ranging from asymptomatic and benign to severe life-threatening ones have been reported to occur at various frequencies. Different uterotonics and vasopressors used during the course of cesarean section under spinal anesthesia have arrhythmogenic potential. The sensory level of spinal anesthesia that is required for cesarean section to be done comfortably is above level T_6_. Block height may be the single most important variable contributing to the occurrence of new conduction delays in otherwise healthy women [6]. Arrhythmia, especially bradycardia is common during hypotension, the most common complication of spinal anesthesia [7].

The etiology of arrhythmia during cesarean section under spinal anesthesia is multifactorial. The effect of arrhythmia and hemodynamic alterations on maternal and immediate neonatal outcome is not yet clear.

This study was thus conducted to evaluate the incidence of different types of arrhythmia during cesarean section under spinal anesthesia and to identify the associated risk factors and its effects on immediate post-delivery neonatal outcomes. The knowledge acquired would help us modify the potential predisposing factors.

## Materials and methods

This prospective observational study was conducted over a period of one year from October 2019 to September 2020 in a tertiary care hospital of north-eastern India. Institute ethics committee approval was taken before commencing the study. All pregnant women of the American Society of Anesthesiologists (ASA) physical status I and II, irrespective of age, weight, and height planned for elective or emergency cesarean section under spinal anesthesia were included in the study. Any women with preoperative electrocardiographic (ECG) abnormality, electrolyte disturbance, cardiorespiratory, renal, hepatic, or thyroid disease, pregnancy-induced hypertension, or any other condition or medications that may influence the occurrence of arrhythmia were excluded from the study. Patients who required conversion to general anesthesia were withdrawn from the study. Exclusion criteria also included cases with any signs of fetal distress and fetus with congenital anomaly.

Informed written consent was obtained from parturients who were recruited in the study. All study participants were preloaded with 500 milliliters of Ringer’s lactate through an 18 gauge intravenous cannula. ECG, non-invasive blood pressure (NIBP), and oxygen saturation (SpO2) were monitored intraoperatively and continued for an hour postoperatively. All women were given spinal anesthesia in the left lateral position following the aseptic technique. Intrathecally, 2 to 2.6 mL of 0.5% hyperbaric bupivacaine was administered at the L_3_-L_4_ interspinal space using a 25 gauge spinal needle. The patients were then positioned supine with left uterine displacement till the appropriate block height was achieved following which the surgeons were notified to begin the surgery. Oxygen at the rate of two liters per minute via nasal prongs was administered until delivery was accomplished. Ringer’s lactate was administered to all the mothers intraoperatively.

Level of block height was assessed after 5, 10, 15, and 20 minutes of spinal anesthesia (by pin-prick sensation). Blood pressure was recorded at 5 minutes interval. Hypotension, a decrease in mean arterial pressure (MAP) by more than 30% was noted. Also, treatment with intravenous mephentermine or ephedrine, if required, was noted along with the dosage. Uterine manipulation (fundal pressure or exteriorization) was also noted.

All episodes of arrhythmia were recorded and correlated with the maximum level of block height, dose of intrathecal bupivacaine used, uterine manipulation, and hypotension. Block height at the time of arrhythmia and any intervention required to terminate the event were also recorded.

After delivery, neonatal parameters were recorded in terms of APGAR score at 1 and 5 minutes, need for cardiopulmonary resuscitation (CPR), and neonatal intensive care unit (NICU) admission in all the mothers with or without arrhythmia and correlated.

All the data collected were entered in Microsoft Excel (Microsoft Corporation, Redmond, WA) and analyzed using Statistical Package for the Social Sciences (SPSS) software version 21 (IBM Corp. Armonk, NY). Continuous variables were analyzed with independent Student’s t-test and the categorical variables compared using Chi-Square test. A P value less than 0.05 was considered statistically significant.

## Results

The results of the study are tabulated in Tables 1-3.

**Table 1:** Demographic variables. SD: standard deviation; SEM: standard error of the mean; BMI: body mass index

Parameters	Arrhythmia group (n=69)			Non-arrhythmia group (n=147)			P value
	Mean	SD	SEM	Mean	SD	SEM	
Age (in years)	28.07	4.673	0.5625	27.13	4.661	0.3844	0.1704
BMI	23.26	2.325	0.2799	23.64	2.469	0.2036	0.2866

 

**Table 2:** Types of arrhythmia and association with maximum block height.

Types of arrhythmia	No. of women (n=69)	Maximum block height										P value
		Above T4 (n=30)			T4-T6 (n=30)					T7-T8 (n=9)		
Sinus bradycardia	42 (60.9%)	22 (52.38%)			17 (40.48%)					3 (7.14%)		<0.0001
		T2=12		T3=10	T4=12		T5=1		T6=4	T7=0	T8=3	
Sinus arrhythmia	15 (21.7%)	8 (53.33%)			5 (33.33%)					2 (13.33%)		<0.0001
		T2=4	T3=4		T4=1	T5=2		T6=2		T7=2	T8=0	
Premature atrial contractions	6 (8.7%)	0			6 (100%)					0		
					T4=3	T5=2		T6=1				
Premature ventricular contractions	3 (4.3%)	0			2 (66.67%)					1 (33.33%)		<0.0001
					T4=1	T5=0		T6=1		T7=1	T8=0	
Supraventricular tachycardia	2 (2.9%)	0			0					2 (100%)		
										T7=1	T8=1	
Mobitz type I	1 (1.4%)	0			0					1 (100%)		
										T7=0	T8=1	

**Table 3:** Arrhythmia and the associated factors and outcome. APGAR: Appearance (skin color), Pulse (heart rate), Grimace (reflex irritability), Activity (muscle tone), and Respiration; NICU: neonatal intensive care unit

Parameters		Arrhythmia group N(%)	Non-arrhythmia group N(%)	P value
Maximum level of block height	Above T4	30 (65.2%)	16 (34.8%)	0.0001
	T4 and below	39 (23%)	131 (67%)	
Drug dosage	≤ 2.2mL	35 (25.5%)	102 (74.5%)	0.010
	> 2.2mL	34 (43.03%)	45 (56.97%)	
Uterine exteriorization	Yes	6 (19.3%)	25 (80.7%)	0.1443
	No	63 (34%)	122 (66%)	
Fundal pressure	Yes	7 (20%)	28 (80%)	0.1151
	No	62 (34.3%)	119 (65.7%)	
Hypotension	Present	30 (17.4%)	142 (82.6%)	<0.0001
	Absent	39 (88.6%)	5 (11.4%)	
APGAR score at 1 min	< 7	8 (27.6%)	21 (72.4%)	0.6726
	≥ 7	61 (32.6%)	126 (67.4%)	
APGAR score at 5 mins	< 7	2 (28.6%)	5 (71.4%)	1.0
	≥ 7	67 (32%)	142 (68%)	
NICU admission	Yes	0	0	
	No	69 (32%)	147 (68%)	

A total of 567 cesarean sections were performed during the study period, of which 216 pregnant women fulfilling the inclusion criteria were included in our study. Out of these 216 women, 69 women (31.9 %) had occurrence of different types of arrhythmia during cesarean section under spinal anesthesia.

Women in both the arrhythmia and non-arrhythmia group were similar with respect to their age and body mass index (BMI) (Table 1).

Among the different types of arrhythmia, the most common was sinus bradycardia (n=42; 60.9%) followed by sinus arrhythmia (n=15; 21.7%), premature atrial contractions (n=6; 8.7%), premature ventricular contractions (n=3; 4.3%), supraventricular tachycardia (n=2; 2.9%), and Mobitz type I (n=1; 1.4%) (Table 2). The overall incidence of bradycardia in patients undergoing cesarean section under spinal anesthesia was 19.4% (42 out of 216). There were two cases of asystole that were preceded by sudden bradycardia.

Women with maximum block height above T_4_ had significantly more arrhythmia than those with lower block heights (P value 0.0001) (Table 3). Also, sinus bradycardia and sinus arrhythmia were significantly associated with rising levels of maximum block height (P value <0.0001 for both) (Table 2). More than half (52.38%) of the sinus bradycardia cases were associated with a block height above T_4 _(Table 2).

Hyperbaric bupivacaine (0.5%) was used in all cases though the doses varied ranging from 2 mL to 2.6 mL. 25.5% of patients who received doses up to 2.2 mL had arrhythmia; however, 43.03% of patients who received dose more than 2.2 mL had some type of arrhythmia and the difference was statistically significant (P value 0.010) (Table 3).

Uterine exteriorization was done in 31 cases, either to look for posterior wall or for uterine repair, of which six cases developed arrhythmia. No statistically significant association was found between them (P value 0.1443) (Table 3). The duration of exteriorization ranged from 40 seconds to 21 minutes depending upon the indication. Five out of six cases developed sinus bradycardia while manipulating the uterus. Two of the five sinus bradycardia cases complained of some discomfort on manipulation and then rapidly succumbed to asystole lasting for 2-3 seconds. Uterine manipulation was halted immediately and stat intravenous injection of 0.6 mg atropine was administered. Both the patients had block height of T_8_ and both were hemodynamically stable prior to the event. Supraventricular tachycardia occurred in one patient on uterine exteriorization with T_8_ level of block. The episode lasted for 5 seconds without any consequence and reversed without any intervention.

Fundal pressure during delivery of the fetus resulted in sinus bradycardia (as low as 45 beats per minute) in seven cases. Block height attained were T_8_ in two cases and T_6_ in five cases. The episodes were self-limiting, resolved without any intervention and no statistically significant association was found between fundal pressure and arrhythmia (P value 0.1151) (Table 3).

Hypotension was recorded in 79.6% of cases (172 out of 216), lasting for 168 seconds to 12 minutes in total duration. In 43.47% (30 out of 69) cases, arrhythmia was preceded by hypotensive episodes, of which 18 were sinus bradycardia, 10 were sinus arrhythmia, and two were premature atrial contractions. A statistically significant association was found between hypotension and arrhythmia (P value <0.0001) (Table 3).

Neonatal outcome in terms of APGAR score at 1 and 5 minutes was similar in both the groups (P value 0.6726, 1.0 respectively) (Table 3). None of the neonates in either group required CPR or NICU admission within an hour of birth.

## Discussion

Spinal anesthesia is the most common type of anesthesia administered during cesarean section, although there are reports of various types of arrhythmia intraoperatively such as bradycardia, ventricular tachycardia, atrioventricular (AV) conduction block, premature contractions, etc. An incidence of arrhythmia of 31.9% was found in our study which was mostly uneventful and benign but a couple of them progressed to asystole or cardiac arrests. Sinus bradycardia was the most common type of arrhythmia (19.4%) in our study. Shen et al. reported an incidence of arrhythmia of 79.52% in patients undergoing cesarean section under spinal anesthesia which was much higher compared to our findings. However, in their study, bradycardia was present in only 6.7% of cases and one of them had loss of consciousness [1]. They found increased parturient age as the most important factor contributing to the occurrence of arrhythmia, however, we found block height, drug doses, and hypotension as the major factors influencing the incidence of arrhythmia under spinal anesthesia during cesarean section (Table 3). In our study, sinus bradycardia was more common in patients who had a maximum block height above T_4 _(Table 2). Block height above T_4_ may inhibit cardio-accelerator fibers (T_1_-T_4_) resulting in unopposed parasympathetic effect on sinoatrial (SA) and AV node with consequent bradycardia. In our study, 18 cases of bradycardia were preceded by hypotension which can be explained by the Bezold-Jarisch reflex where reduced venous return results in reflex bradycardia. Nishikawa et. al. also reported a case where changing position from Trendelenburg to supine resulted in bradycardia and asystole secondary to reduced venous return [5]. Geffin et. al. published 13 cases of bradycardia and asystole of which 12 cases were performed under spinal anesthesia and one under epidural anesthesia. He pointed out a reflex associated with low right cardiac filling as the probable cause [8]. Carpenter et. al. also described block height T_5_ and above as an important determinant for the development of bradycardia under spinal anesthesia and in most of the cases they were benign [6].

Uterine manipulation in the form of exteriorization is not uncommon during the cesarean section for uterine repair or inspection of the posterior wall. Two cases in our study had sudden bradycardia during uterine manipulation which progressed to asystole, however, the episodes were reversed on stopping the uterine manipulation and did not last for more than 2 to 3 seconds. Both the women had block height of T_8_ and complained of anxiety and discomfort during manipulation. Pain, anxiety, or emotional stress may result in such episodes of vasovagal syncope. Many cases have been reported where hemodynamic instability followed placental expulsion, traction, or inversion of the uterus. Vasovagal reflex via afferent nerve stimulation due to infundibulopelvic ligament stretching may be responsible for such episodes [9]. Proper attention should be given to patient’s discomfort and supplemental analgesics administered accordingly. Closed vigilance during this period is necessary for its early detection and timely intervention.

Kalra et. al. reported an episode of benign, asymptomatic Mobitz type I block in a patient without any underlying heart disease or conduction block undergoing cesarean section under spinal anesthesia. The maximum block height was T_5_ and he cited parasympathetic overactivity secondary to cardiac sympathetectomy and reduced venous return as the cause [10]. In our study, one patient developed Mobitz type I block around 50 minutes following spinal anesthesia which lasted for 12 seconds without any symptoms and required no intervention. However, here the block height was T_8_ and there was no uterine maneuver or complaint of pain or discomfort prior to the arrhythmic event. Compared to Mobitz type II which arises at infra-nodal level, type I block usually arises at AV nodal level with lesser chances of progression to complete heart block. Type I block may occur due to multiple organic causes, however, may also occur in 1-2% of the healthy population without any consequence. The underlying reason of this episode in our patient was not clear.

Drug dosage or volume may influence the incidence of arrhythmia secondary to higher level of block and also hypotension. By reducing the dose of an anesthetic drug, arrhythmia can be minimized but at the cost of supplemental analgesic requirement and risk of conversion to general anesthesia. Ginosar et. al. suggested against the use of low dose bupivacaine for spinal anesthesia in case of emergency cesarean section due to the higher risks of failure and delayed onset [11,12]. There has not been any recommended optimum dose of local anesthesia for adequate level of block and patient satisfaction with minimum hemodynamic disturbances. The incidence of inadequate sensory block was very high when doses lower than 10 mg were used in an attempt to reduce hemodynamic adverse effects [13]. Considering the association of lower dose of local anesthetic agents (≤ 2.2 mL) with lesser incidence of arrhythmia (P value <0.01) found in our study (Table 3), it would be wise to optimize the drug dose which would achieve adequate sensory level of block for surgery without increasing the risks of arrhythmia.

The effect of different systemically administered drugs on the occurrences of arrhythmia under spinal anesthesia during cesarean section is difficult to evaluate because of multiple overlapping factors. Though there is a theoretical possibility of arrhythmia following oxytocin infusion, it is rarely reported [14]. And at the usual dose, there is less arrhythmia with ephedrine administration. Ergometrine may however cause AV block and ventricular tachycardia along with coronary vasospasm [15]. Different drugs were used during the hypotensive episodes in our study. In none of the cases, arrhythmia was found to be a direct consequence of administration of the drugs as no arrhythmia occurred within the duration between drug administration and expected peak dose effect. However, pharmacokinetics and dynamics of these drugs also vary from patient to patient and so it is very difficult to assess the possibility of drug effect on arrhythmia as other factors overlap too.

Blockade of cardio-accelerator fibers resulting from high level of block may cause hypotension and bradycardia at the same time making it difficult to predict if one is the cause or consequence of another in such clinical conditions. In 43.47% of the cases of arrhythmia in our study, hypotension has been found to precede arrhythmia which could be due to reflexes secondary to the reduced venous return and hypotension. A proper preloading or co-loading and frequent monitoring of blood pressure especially after commencement of spinal anesthesia or following position changes along with a prompt correction would help prevent the development of such events [16].

Women with antenatal arrhythmia may have neonatal effects in terms of intrauterine growth retardation or placental abruption [17]. However, the effect of intraoperative arrhythmia on immediate neonatal outcome during cesarean section under spinal anesthesia has not been mentioned in the literature. In our study, two cases of sudden asystole of less than 3 seconds occurred without any affect on the neonatal outcome. The possibility of adverse immediate neonatal outcome in cases of intraoperative arrhythmia causing life-threatening hemodynamic fluctuation cannot, however, be ruled out based on the results of our study as majority of the arrhythmia were self-limiting and did not require any intervention.

## Conclusions

The occurrence of arrhythmia during cesarean section under spinal anesthesia is very common but may rarely be life-threatening. Keeping the block height between T_4 _and T_6_, using a lower dose of bupivacaine and early detection and management of hypotension may reduce the incidence of arrhythmia. Also, pain and discomfort during uterine manipulations, especially when the block height recedes below T_8_ may lead to sudden bradycardia and cardiac asystole which can be prevented by paying attention to patient’s subjective symptoms and intense monitoring of pulse and ECG with prompt necessary intervention. The occurrence of intraoperative arrhythmia, however, does not adversely affect the immediate neonatal outcome.
